# A Brief Workplace Training Program to Support Help-Seeking for Mental Ill-Health: Protocol for the Helipad Cluster Randomized Controlled Trial

**DOI:** 10.2196/55529

**Published:** 2024-05-24

**Authors:** Philip J Batterham, Amelia Gulliver, Cassandra Heffernan, Alison L Calear, Aliza Werner-Seidler, Alyna Turner, Louise M Farrer, Mary Lou Chatterton, Cathrine Mihalopoulos, Michael Berk

**Affiliations:** 1 Centre for Mental Health Research College of Health and Medicine The Australian National University Acton Australia; 2 Black Dog Institute University of New South Wales Sydney Australia; 3 Deakin University School of Medicine IMPACT, The Institute for Mental and Physical Health and Clinical Translation Geelong Australia; 4 School of Public Health and Preventive Medicine Monash University Melbourne Australia; 5 Orygen, The National Centre of Excellence in Youth Mental Health Centre for Youth Mental Health, Florey Institute for Neuroscience and Mental Health and the Department of Psychiatry The University of Melbourne Melbourne Australia

**Keywords:** help seeking, mental health, workplace, employee, implementation, internet, psychiatry, psychology, mobile phone

## Abstract

**Background:**

Most people with mental health problems do not seek help, with delays of even decades in seeking professional help. Lack of engagement with professional mental health services can lead to poor outcomes and functional impairment. However, few effective interventions have been identified to improve help-seeking in adults, and those that exist are not widely implemented to deliver public health impact. Co-designing interventions with people with lived experience of mental ill-health and other relevant stakeholders is critical to increase the likelihood of uptake and engagement with these programs.

**Objective:**

This study aims to (1) test the effectiveness of a co-designed help-seeking program on increasing professional help-seeking intentions in employees in a workplace setting; (2) determine whether the program reduces mental illness stigma and improves help-seeking intentions and behavior, mental health literacy, mental health symptoms, and work and activity functioning relative to the control condition; (3) explore factors that facilitate broader implementation of the co-designed program; and (4) explore the cost-effectiveness of the co-designed program compared to the control condition over 6 months.

**Methods:**

A 2-arm cluster randomized controlled trial will be conducted (target sample: N=900 from 30 to 36 workplaces, with n=25 to 35 participants per workplace). The trial will compare the relative effectiveness of an enhanced interactive program (intervention condition) with a standard psychoeducation-alone program (active control condition) on the primary outcome of professional help-seeking intentions as measured by the General Help-Seeking Questionnaire. Secondary outcomes include the impact on mental illness stigma; mental health literacy; help-seeking attitudes and behavior; work and activity functioning; quality of life; and symptoms of mental ill-health including depression, anxiety, and general psychological distress.

**Results:**

Facilitators of and risks to the trial are identified and addressed in this protocol. Recruitment of workplaces is scheduled to commence in the first quarter of 2024.

**Conclusions:**

If effective, the program has the potential to be ready for rapid dissemination throughout Australia, with the potential to increase appropriate and efficient service use across the spectrum of evidence-based services.

**Trial Registration:**

Australian New Zealand Clinical Trials Registry (ANZCTR) ACTRN12623000270617p; https://www.anzctr.org.au/Trial/Registration/TrialReview.aspx?id=385376

**International Registered Report Identifier (IRRID):**

PRR1-10.2196/55529

## Introduction

### Background

In a 12-month period, 16.8% of Australian adults will experience an anxiety disorder and 4.6% will experience a major depressive episode [1]. Despite the high prevalence of common mental disorders, most people with a mental health problem will not seek help from a health professional [1]. Among those who seek help, delays of years or decades to seeking professional help are common [2,3]. The lack of engagement with professional services may lead to poorer outcomes in both mental health symptoms and functional impairment [4] and productivity loss [5-8]. While structural factors such as service accessibility impact help-seeking, we also know that individual factors such as the stigma of mental illness, the stigma of help-seeking, poor mental health literacy, and high self-reliance reduce the likelihood of an individual seeking help when experiencing symptoms of mental ill-health [9,10].

### Mental Health Promotion Programs

Promotion programs that are universally delivered (ie, provided to all people in a population, regardless of risk or symptom level) have the potential to improve mental health. Such programs often involve targeting key barriers to help-seeking, such as knowledge or mental health literacy (including how, where, and when to seek help), and reducing stigmatizing attitudes toward help-seeking [11]. Facilitating pathways to professional help is critical, as treatment delays from the onset of symptoms can be as long as 8 years for depression and up to 23 years for anxiety disorders, leading to potentially significant and avoidable disability [3]. Mental disorders such as depression and anxiety can follow a chronic and treatment-resistant course in the absence of early intervention [12]. Therefore, there is increasing recognition of the need for promotion and prevention approaches.

Much of the evidence for the effectiveness of universal prevention programs on knowledge and mental health outcomes comes from adolescent settings [13,14], typically showing modest effects that may have a significant impact when delivered at scale. However, >50% of anxiety disorders and 75% of mood disorders emerge in adulthood [15], and they have high rates of relapse [16]. Workplaces offer an ideal environment to deliver prevention programs due to the significant proportion of adults in paid employment and the motivation among employers to ensure their employees remain well and productive. Prevention programs for adults delivered in the workplace typically focus on reducing emerging or subthreshold symptoms of depression, anxiety, or distress, with very modest effects for universally delivered programs [17]. A promising alternative approach is to promote help-seeking for distress by increasing knowledge about mental health and pathways into care, along with reducing stigmatizing attitudes about mental ill-health [18,19]. However, very few help-seeking promotion programs have been evaluated in general adult populations, with just 2 focusing on workplaces [20] and neither being implemented broadly. Consequently, there may be untapped opportunities for developing and implementing evidence-based mental health promotion programs within workplaces that ultimately lead to greater support for adults who experience mental distress.

### Co-Design and Online Delivery Modes

The COVID-19 pandemic has dramatically accelerated the need for mental health programs [21], particularly those delivered distally [22]. However, few effective programs have been identified for improving help-seeking in adults [23], and existing interventions are not widely implemented to deliver public health impact [19,24]. Web-based mental health interventions are cost-effective [25] and can be widely disseminated [26], but they can have high dropout rates, increasing the need for co-design to improve acceptability [27]. There is increasing recognition of the expertise of people with lived experience of mental health problems in research, and the important insights end users of a product or service can bring to the research process, by improving translation, real-world impact, and responsive practice [28]. Co-design involves partnering with end users during all stages of designing and developing a product or service. Using this method enhances the potential that the service can be tailored to the target population, creating a strong potential to foster a sense of empowerment and ownership, which in turn may improve the uptake of and engagement with the service [27].

### This Study

Programs that promote help-seeking intentions and behaviors can reach large proportions of people when delivered at scale within workplaces. This paper is a protocol for a cluster randomized controlled trial (RCT) that will provide a robust evaluation of the effectiveness and cost-effectiveness of a novel co-designed program for improving help-seeking in workplaces. Furthermore, this study examines potential factors that may impede or enhance the subsequent delivery and implementation of the program.

### Aims

The aims of this study are as follows:

1. To test the effectiveness of a co-designed help-seeking program (the Helipad program) on increasing professional help-seeking intentions (primary outcome) in employees in a workplace setting at posttest (primary end point) and the 6-month follow-up
2. To determine whether the program reduces mental illness stigma and improves help-seeking attitudes and behavior, mental health literacy, mental health symptoms, quality of life, and functioning (secondary outcomes) relative to the control condition at the 6-month follow-up
3. To explore factors that facilitate broader implementation of the co-designed program
4. To explore the cost-effectiveness of the co-designed program compared to the active control condition from the health sector and societal perspectives at the 6-month follow-up

### Hypotheses

To investigate the abovementioned aims, we propose the following hypotheses.

#### Primary Hypothesis (Aim 1)

- Hypothesis 1: Help-seeking intentions will be higher in the Helipad co-designed interactive program condition relative to the control condition (standard psychoeducation only) at posttest.

#### Secondary Hypotheses (Aim 2)

- Hypothesis 2: Mental illness stigma will be lower and mental health literacy and help-seeking attitudes will be higher in the Helipad co-designed interactive program condition relative to the control condition (standard psychoeducation only) at posttest.
- Hypothesis 3: Help-seeking behavior will be greater in the Helipad co-designed interactive program condition among people with elevated symptoms of depression or anxiety relative to the control condition (standard psychoeducation only) at the 6-month follow-up.
- Hypothesis 4: Symptoms of mental ill-health will be lower and quality of life and functioning will be higher in the Helipad co-designed interactive program condition relative to the control condition (standard psychoeducation only) at the 6-month follow-up.

#### Exploratory Hypotheses (Aim 3)

We do not have any specific hypotheses for factors that facilitate broader implementation of the program as this is exploratory; furthermore, we will use interviews with participants to explore this aim.

#### Exploratory Hypotheses (Aim 4)

The Helipad program will be cost-effective relative to the active control (psychoeducation) at the 6-month follow-up from health sector and societal perspectives relative to the willingness-to-pay thresholds identified by the Productivity Commission report on mental health [29].

## Methods

### Trial Design

A 2-arm cluster RCT will test whether the Helipad co-designed interactive program increases help-seeking intentions in employees in workplaces more than the standard psychoeducation-alone program. Workplaces will be randomly allocated using minimization processes to either (1) the active intervention condition comprising the Helipad program or (2) an active control condition (a web-based time-matched standard psychoeducation program). Help-seeking intentions will be assessed at the individual level accounting for clustering by workplace. Self-report assessments will be conducted on the web at preintervention, immediately postintervention, and at the 6-month follow-up. This study protocol addresses the Standard Protocol Items: Recommendations for Interventional Trials (SPIRIT) checklist [30].

**Figure 1:**
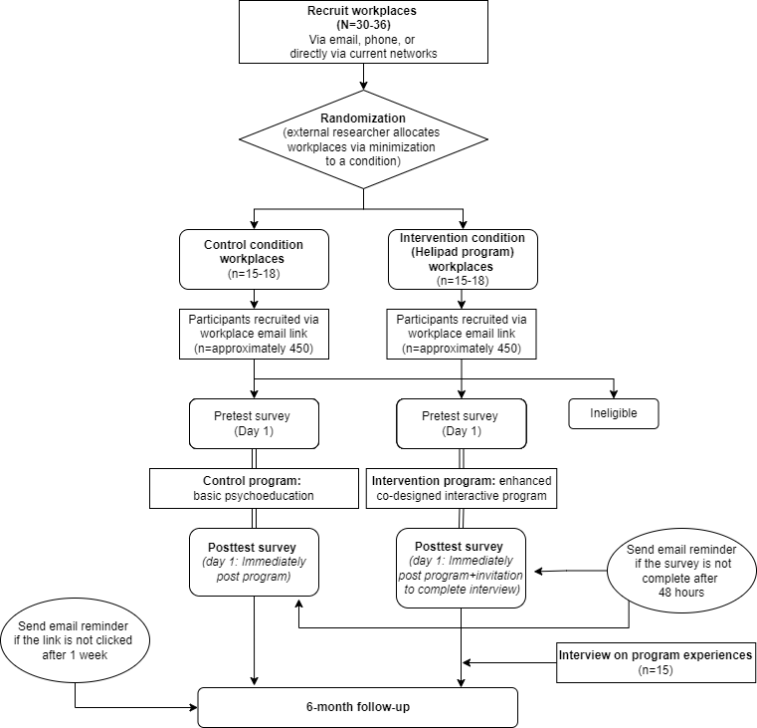
Cluster randomized controlled trial flowchart for the Helipad brief workplace training program to support help seeking for mental ill-health.

The flow of participants through the study is presented in Figure 1.

### Interventions

#### The Helipad Program

A co-design working group was convened to develop the program from May to October 2023. Two authors, who identify as lived experience researchers (AG and CH), facilitated and led the co-design working group. The program was developed using principles of community-based co-design research, which are particularly appropriate in implementation research that focuses on the relevance and uptake of interventions within and across organizations [31]. The co-design group comprised 9 team members from the community. In addition, 2 web development team members were consulted during the later stages of the co-design working group’s activities. All 9 community co-design team members were currently employed, and almost all identified as belonging to >1 group (consumers: 6/9, 67%; carers: 5/9, 56%; managers: 2/9, 22%; human resources staff: 2/9, 22%; and clinician: 1/9, 11%). The co-design process is outlined in detail in a separate paper (Gulliver et al, forthcoming).

The resulting co-designed program, called Helipad, is brief and takes approximately 20 minutes to complete. Brevity was essential to ensure that the information provided could be easily digestible and did not form a barrier to completing the program.

The Helipad program consists of 5 interactive modules addressing the core issues described by the co-design group as being important to facilitating help-seeking in workplaces (Textbox 1). The modules have been designed to be highly interactive, including clickable diagrams, videos of health professionals and people with lived experience, and interactive quizzes.

**Interactive modules of the Helipad program.**

**Modules**

1. Recognizing symptoms: explores signs and symptoms via a traffic light model (red, orange, and green indicators) of mental health. This module comprises an interactive map of the body that explains how we can recognize signs, symptoms, or indicators in our minds and bodies that tell us we might need to pay attention to our mental health and suggests what we can do about it.
2. Seeking support: answers questions regarding how, where, and from which health professionals to seek help for mental health and how each provides assistance. Moreover, it demystifies the process of seeking help by providing a specific example pathway (ie, accessing a psychologist with or without a referral from a general practitioner [GP]) and 3 videos of health professionals (Employee Assistance Program, psychologist, and GP) explaining what they do to support people’s mental health.
3. Treatment options: provides information regarding evidence-based treatment options including psychological treatment, medication, exercise, and lifestyle treatments for common mental health problems, such as depression and anxiety. The psychological strategies were focused primarily on strategies approved by the Australian Government Medicare Benefits Schedule (MBS [32]), which are the most accessible evidence-based treatments available through the MBS, such as cognitive behavioral therapy (CBT), with medical and lifestyle treatments suggested by members from the co-design group. Furthermore, this module contains a practical example of a specific CBT strategy called “cognitive restructuring,” works and an exercise on identifying people who may support them during challenging times.
4. Helping others: explores how to help others in the workplace who may disclose their experiences of mental ill-health and provides some examples about how different responses to this disclosure can affect the outcome for the person experiencing a mental health issue. In addition, this module contains a video of a manager discussing their experience of an employee who disclosed their mental health issue.
5. Supportive workplaces: addresses stigmatizing attitudes toward mental ill-health, help-seeking, and disclosure in the workplace. This module contains 3 videos of peers describing their experiences of disclosing in the workplace, myth-busting information in the form of a quiz on common mental disorders of depression and anxiety, and information targeting concerns about disclosure of mental ill-health in the workplace.

#### Active Control Program

The active control condition was chosen to maximize participant and organizational engagement in the trial and provide a strong test of the efficacy of the active ingredients of the Helipad program. The program comprises 5 time-matched standard written psychoeducation modules about mental health and well-being. The modules comprise written content from the National Institutes of Health News in Health newsletters [33] that was minimally edited by the research team (eg, to combine content from separate papers on the same topic). Written content focused on education on signs and symptoms of common mental health conditions of depression and anxiety disorders and how aspects of our lives (ie, sleep, exercise, relationships, and stress) impact our mental health. However, this program did not contain detailed information regarding treatment options, content explicitly targeting stigma reduction (eg, contact via peer stories), or how or where to seek help from health professionals. Furthermore, the content was not co-designed, is minimally interactive, and does not provide targeted information about help-seeking processes. Psychoeducation has been shown to increase mental health literacy and may have modest effects on reducing stigma but does not typically translate into changes in behavioral intentions such as help-seeking [24].

The modules are as follows:

1. Sleep and well-being: contains information regarding sleep, including sleep stages, sleep quality and duration, and how lack of sleep affects our overall health and well-being.

2. Exercise and physical health: provides information on how exercise is beneficial for health and well-being and tips on how to increase overall physical activity.

3. Mental health: provides information on depression and anxiety disorders, including signs and symptoms and how they can be treated.

4. Social relationships and health: explores how social relationships affect our health in both positive and negative ways and how social support may buffer stress.

5. Stress and health: provides information regarding stress, including the impact of stress on our physical and mental health and how we can reduce stress in daily life.

### Recruitment Procedures and Randomization

#### Workplace Recruitment

Recruitment of workplaces is expected to be conducted using our existing networks through the The Mental Health Australia General Clinical Trials Network. We plan to recruit approximately 30 to 36 workplaces, with 25 to 35 participants per workplace to meet our total sample size target of 900 adult participants. Workplaces will be eligible if they (1) report having at least 50 employees to ensure that each cluster is viable and (2) have an Employee Assistance Program (EAP) available for their staff to ensure that employees experiencing mental health concerns can access effective and immediate care. Any industry of work is eligible for inclusion. A mixture of general and high-risk workplaces (ie, those likely to be exposed to situations that may impact mental health, such as health care settings) initially in the Australian Capital Territory, New South Wales, and Victoria are being targeted. Recruitment is highly feasible based on previous public health trials conducted by the team (N=849 to 2773 [34-36]), and our universal cluster-based trials have typically recruited high numbers (eg, N=1477 [37] and N=1633 [38]).

We will invite workplaces through multiple channels, including directly via an email invitation addressed to the “key representatives” from a workplace such as managers or staff in charge of human resources, via telephone calls, and via invitation emails to employees in our existing networks. We will contact the workplace key representatives who express interest to determine organization characteristics and eligibility for the study.

#### Randomization

Given the cluster design, we will use a minimization approach to randomization [39]. Minimization is a type of randomization where the first participant (or cluster) is randomized and subsequent selections are allocated in a way that minimizes the imbalance on specific factors between groups. This method is helpful for a design with few participants (or clusters) and has the advantage of making each condition more closely matched on key factors across the trial. To implement this, we will use a preprogrammed computer algorithm that will assign each new workplace recruited into the trial into a condition. This process will aim to balance across conditions on the following key workplace characteristics: (1) workplace type (office-based vs non–office based), (2) organization size (<200 vs >200 employees), and (3) location (Australian Capital Territory or New South Wales or Victoria vs other Australian state or territory). Once a workplace has been allocated a condition, we will discuss with their key representative what participation in the trial involves and provide them with an email or SMS text message (depending on their preferred communication with employees) with an embedded link to their assigned study recruitment website. We expect the text to read as follows:

Would you like to participate in a research study testing a new resource for supporting employee wellbeing? Learn more about your mental health and wellbeing by completing a survey and a brief online program today: <link>.

We will ask the workplaces to send invitations to all their employees themselves, with each workplace determining who from within their organization will send this invitation. This process is designed to increase ecological validity, as the goal for the program is for it to be delivered by workplaces, we expect this to be a minimal burden (ie, 1 or 2 SMS text messages or emails) on the workplace administration staff tasked with sending invitations.

#### Participant Recruitment and Informed Consent

Participants will be recruited on the web through participating workplaces. To be eligible for the trial, potential participants must fulfill the following criteria:

- Be an employee in a participating Australian workplace
- Be aged ≥18 years, living in Australia, and fluent in reading and understanding English
- Have access to a device (eg, desktop, laptop, tablet, or smartphone) and a reliable internet connection

If participants complete the abovementioned screening criteria and are not eligible, they will be informed and provided with a list of relevant help-seeking resources.

After being assigned a condition, the workplace will send a brief email or SMS text message to their employees inviting them to participate in the trial (refer to the Randomization section for an example). The email or SMS text message will contain a link to the web page containing information about the study. Then, the employees who click the link provided by their employers will be taken to the information and consent web-based page, where they will be invited to read the information that the study and provide their consent to participate in the study on the web. This process will be confidential, and their work organization will not be informed of who among their employees has consented to participate in the trial. Participants can complete the preintervention, program, and postintervention survey in a single session. Furthermore, participants can save their progress at the bottom of the screen if they wish and re-enter the survey or program at the last point they left off by reaccessing the link from their email or SMS text message. In addition, they will be sent reminders to complete the program if they have not done so within the expected time frames (Figure 1).

### Outcomes

#### Estimand

In workplace employees, what is the between-group difference for the Helipad co-designed web-based program compared with the standard psychoeducation web-based program as measured by change from preintervention to immediately postintervention on help-seeking intentions on the General Help-Seeking Questionnaire (GHSQ)?

#### Primary Outcome

Help-seeking intentions (hypothesis 1) will be assessed based on increased intentions to seek help on the GHSQ completed at posttest.

#### Secondary Outcomes

Effectiveness will be compared between the Helipad co-designed interactive program condition and the standard psychoeducation web-based program condition for mental illness stigma, mental health literacy, and help-seeking attitudes (hypothesis 2), which will be assessed, respectively, based on reduced mental health stigma (Stigma and Self-Stigma Scales [40]), increased mental health literacy (Anxiety Literacy Questionnaire [A-Lit] and Depression Literacy Questionnaire [D-Lit] composite measure [41,42]), and increased help-seeking attitudes (Attitudes Toward Seeking Professional Psychological Help Scale-short form [43]) over time relative to the control condition.

In addition, we will examine the comparative effectiveness of the Helipad co-designed interactive program condition and the standard psychoeducation web-based program condition on reducing symptoms of mental ill-health and increasing quality of life and functioning (hypothesis 4). This will be determined based on reduced symptoms over time of depression (Patient-Reported Outcomes Measurement Information System [PROMIS] Depression short form scale [44]), anxiety (PROMIS Anxiety short form scale [44]), and general psychological distress (Distress Questionnaire-5 [DQ5] [45]) and increased scores on the Recovering Quality of Life (ReQoL-10) Questionnaire [46] and increased work and activity functioning (Work Productivity and Activity Impairment [WPAI] questionnaire [47]).

Finally, the relative effects of the program on increasing help-seeking behavior (hypothesis 3) will be assessed by the proportion of participants who reported seeking help from a professional source (defined as a psychologist or counselor, a psychiatrist, a family doctor or general practitioner [GP], or an EAP) in the past 6 months. We anticipate that this will be greater in the Helipad co-designed interactive program group among people with elevated symptoms of depression or anxiety relative to the control condition (standard psychoeducation only).

The primary end point for changes in mental health literacy is the posttest assessment, while for the other secondary outcomes, the 6-month follow-up assessment will be the primary end point as these outcomes take longer to elicit change.

### Data Analysis

An intention-to-treat approach to account for missing data of the mixed model repeated measures ANOVA (continuous data) or mixed effects logistic regression (binary data) will be used, adjusting for intraclass correlation to account for clustering effects within each site. Mixed models include all available data and account for within-individual variability, including those participants with missing data points, under the missing-at-random assumption [48].

Exploratory analyses will examine evidence for moderators or mediators of outcome and will be reported separately from the primary and secondary outcomes. Implementation outcomes at 6 months will be separately examined using description statistics, including *t* tests (2-tailed). The economic analysis will examine cost-effectiveness from the health sector and societal perspectives. The cost of the Helipad intervention and the active control program will be estimated using microcosting methods. Service use data will be limited in this study to health care professional visits or services accessed (internet program and helpline) specifically for mental health. The additional costs of health care resource use will be operationalized as the number of contacts with health professionals collected on the Actual Help-Seeking Questionnaire [49] (psychologist or counselor, psychiatrist, family doctor or GP, EAP, phone helpline, and internet-based treatment program). Lost productivity will also be evaluated in societal perspective costs as assessed through the WPAI questionnaire [47] and valued using the human capital approach. The ReQoL-10 Questionnaire [46] will be used to calculate utility values and quality-adjusted life years (QALYs) using the area under the curve method. Differences in costs between trial groups will be compared to the differences in QALYs through an incremental cost-effectiveness ratio (ICER) because of the inherent value-for-money connotations associated with cost-effectiveness ratios using QALYs. Additional ICERs using secondary outcomes will be calculated where the Helipad intervention is found to have an effect. Nonparametric bootstrapping will be used to determine CIs for ICERs.

In addition, we will report proportions of participants who improve, decline, and do not change on outcome measures. Automatically collected aggregate use data will also be analyzed, including information on how many participants access each page, location, average time on each page, video view count data, and clicks to external links.

### Trial Delivery

The trial will be delivered using a custom-designed website that will contain the 2 web-based programs. This website will allow self-report assessments, intervention materials, and email reminders to be delivered seamlessly to the participants. Furthermore, the website will collect and securely house the survey data collected. To streamline access and reduce the need for participants to create log-ins while maintaining security and confidentiality, participants will only need to provide an email to access the program.

If surveys or the program are discontinued, participants will be emailed a single reminder (at 48 hours if they discontinue any part of the preintervention, program, and postintervention survey and 1 week for the 6-month follow-up survey) with a unique link to access the program from their email account to continue from where they left to complete that part of the study. We expect most participants will complete the preintervention, program, and postintervention surveys in one 40-minute sitting. As such, we do not anticipate that these reminders will be needed frequently; however, given that people could discontinue the program or surveys, we have a method to reconnect them with the study.

### Data Collection

#### Overview

Participants who agree to participate in the trial by clicking the link will be asked to provide an email address and complete a brief 10-minute baseline questionnaire. Following completion of the baseline questionnaire, participants will immediately be given access to the web-based program (approximately 20 minutes), which will be followed immediately by the 10-minute posttest questionnaire survey. The 10-minute follow-up survey link will be sent to participants via email 6 months after completion of their allocated condition.

#### Assessments and Blinding

Table 1 presents the trial measures and assessment time points. Assessments will occur at baseline (pretest), posttest, and 6 months after the program is completed. The trial will be triple blinded. Participants will be blinded as far as possible to the condition; they will only be informed that their workplace has been randomized to receive one of two programs: (1) an enhanced interactive mental health education program or (2) a standard written psychoeducation program. We will not provide participants with information about which program is expected to be the most effective. The statistician who will perform the analyses will be blinded to condition allocation (triple blind). Finally, assessments will also be blinded, as they rely on participants independently completing self-report measures on the web.

**Table 1:** Assessment time points for the Helipad cluster randomized controlled trial of a brief workplace training program to support help-seeking for mental ill-health.

Construct	Measure	Presurvey	Postsurvey	6-month follow-up survey
Gender	What is your gender?	✓		
Age	What is your age?	✓		
Language spoken at home	What language do you usually speak at home?	✓		
Ethnicity	How would you describe your ethnicity?	✓		
Education	What is the highest level of education that you have completed?	✓		
Employment status	What is your current employment status?	✓		
Industry	What industry are you employed in?	✓		
Help-seeking intentions	GHSQ^a^	✓	✓	✓
Help-seeking attitudes	ATSPPH-SF^b^	✓	✓	✓
Help-seeking behavior: service use type and quantity	AHSQ^c^	✓		✓
Mental illness stigma	(SASS^d^-modified)	✓	✓	✓
Mental health literacy	D-Lit^e^ or A-Lit^f^ composite	✓	✓	✓
Depression symptoms	PROMIS^g^ Depression-Short Form	✓		✓
Anxiety symptoms	PROMIS Anxiety-Short Form	✓		✓
General psychological distress	DQ5^h^	✓		✓
Work and activity impairment	WPAI^i^	✓		✓
Quality of life	ReQoL-10^j^	✓		✓
Adverse events	Did you have any unexpected or negative experiences that might be related to completing this program?		✓	✓
Intervention acceptability	AIM^k^		✓	
Intervention appropriateness	IAM^l^		✓	
Intervention feasibility	FIM^m^		✓	
Debriefing	Explanation of study			✓
Interview	Interest in an interview		✓	

^a^GHSQ: General Help-Seeking Questionnaire.

^b^ATSPPH-SF: Attitudes Toward Seeking Professional Psychological Help Scale-short form.

^c^AHSQ: Actual Help-Seeking Questionnaire.

^d^SASS: Stigma and Self-Stigma Scales.

^e^D-Lit: Depression Literacy Questionnaire.

^f^A-Lit: Anxiety Literacy Questionnaire.

^g^PROMIS: Patient-Reported Outcomes Measurement Information System.

^h^DQ5: Distress Questionnaire-5.

^i^WPAI: Work Productivity and Activity Impairment.

^j^ReQoL-10: Recovering Quality of Life-10.

^k^AIM: Acceptability of Intervention Measure.

^l^IAM: Intervention Appropriateness Measure.

^m^FIM: Feasibility of Intervention Measure.

### Measures

#### Demographic Characteristics

These demographic characteristics are planned for assessment at preintervention: gender (male, female, nonbinary, different term, or other), age (18 to 25, 26 to 35, 36 to 45, 46 to 55, 56 to 65, or >66 years), ethnicity (open ended: “how would you describe your ethnicity”), language spoken at home (English only, English and another language, or another language only), level of education (primary school, some secondary school or year 10 equivalent, year 12, Certificate level I-IV, Diploma or Associate degree, Bachelor degree, Graduate Diploma or Graduate Certificate, Master degree, or Doctoral degree), employment status (eg, full time, part time or casual, unemployed, or not currently working due to studying or maternity leave), and work industry (business, consultancy, or management; accountancy, banking, or finance; charity and voluntary work; creative arts or design; energy and utilities; engineering or manufacturing; environment or agriculture; health or social care; hospitality or events; computing or Information Technology; law; law enforcement and security; leisure, sport, or tourism; marketing, advertising, or public relations; media or digital; property or construction; public services or administration; recruitment or human resources; retail or sales; science or pharmaceuticals; teacher training or education; and transport, logistics, or other).

#### Help-Seeking Intentions (Primary Outcome)

Help-seeking intentions will be measured by the intentions scale of the GHSQ [50]. The scale is an index, which for this study comprised 11 items measuring intentions to seek help “if you were having a personal or emotional” problem. Consistent with the GHSQ as a flexible index of help-seeking sources, we modified the scale to group nonprofessional informal sources together, that is, “partner, friend, parent, other relative/family member.” Furthermore, we added “Employee Assistance Programs,” and “internet-based treatment programs” to the potential sources of help and separated mental health professionals (ie, psychologists or counselors vs psychiatrists) to allow for economic analysis. Each source of help is rated on a 7-point Likert-type scale ranging from 1 (extremely unlikely) to 7 (extremely likely), with a midpoint at “unsure.” Item scores range from 1 to 7, with higher scores indicating greater intentions to seek help from that particular source.

#### Help-Seeking Attitudes

The help-seeking attitudes of participants will be measured using a 5-item updated version [51] of the Attitudes Toward Seeking Professional Psychological Help Scale-short form [43]. The 5 items retained were the positively-worded items of 1, 3, 5,6, and 7 and were selected based on factor loadings [52,53]. The scale asks respondents to indicate on a 4-point Likert-type scale (0=disagree and 3=agree) to what extent they agree with statements regarding psychological treatment (eg, “If I was having personal or emotional problems, the first thing I would do is seek professional help”). Scores are summed to produce a possible range of 0 to 15, with higher scores indicating more positive attitudes toward seeking professional help. Both the 5-item modified short form (Cronbach α=0.72) [52] and the 10-item scale have demonstrated sound psychometric properties [43,51,54].

#### Help-Seeking Behavior

Help-seeking behavior will be assessed by the proportion of participants reporting seeking help from a professional help-seeking source in the past 6 months on the Actual Help-Seeking Questionnaire [49]. This questionnaire consists of 10 items measuring self-reported help-seeking behavior. Participants are provided a list of help-seeking sources (matched to the abovementioned list for the intentions scale) and asked to indicate where, if any, they have “gone to for advice or help in the past 6 months for a mental health problem*.*” Furthermore, participants can indicate if they had seen someone else not listed, that they had not spoken with anyone about their problems, or that they had not experienced any problems. The number of visits to each health professional (psychologist or counselor, psychiatrist, family doctor or GP, and EAP) or the number of times a service was accessed (internet program and helpline) will also be assessed. Items from this scale specifying the number of contacts with each health professional or service will be used for the economic analysis.

#### Mental Health Literacy

Mental health literacy will be assessed using a bespoke measure generated by combining items from the D-Lit [41] and A-Lit [42]. The scale comprises 11 items (5 from the D-Lit questionnaire, 5 from the A-Lit, and 1 new item) that ask respondents to indicate their knowledge on a scale of true, false, or do not know for statements regarding the symptoms and treatment of depression and anxiety. Example items include “Depression does not affect your memory and concentration” (false) and “Generalized anxiety disorder is a common cause of workplace disability” (true). The new item added for this study was “Avoiding professional help for my mental health is unlikely to have an effect on my health long-term” (false). The original scales have good internal consistency and reliability, and scores on the bespoke measure will range from 0 to 11 (correct response=1 point), with higher scores indicating higher literacy [41,42].

#### Mental Illness Stigma

To measure stigma, we adapted the “stigma to others” scale from the Stigma and Self-Stigma Scales for attitudes toward mental health problems [40]. We generated a bespoke 4-item scale by retaining 4 of the 6 original items and slightly altering the wording (eg, changing mental disorder to “mental illness” for clarity and removing the word “suffering”). One item was removed due to a low factor loading of 0.32, and another item was double barreled and duplicated another item; therefore, we retained the item with the higher factor loading, leaving 4 items rated on a 5-point scale from 0=strongly disagree to 4=strongly agree: “Employees with a mental illness are less reliable than other employees,” “People with a mental illness are weak,” “People with a mental illness should just “snap out of it,” and “People with a mental illness are not really ill.” The original stigma to others scale demonstrated good validity and internal consistency (Cronbach α=0.71) [40]. Scores range from 0 to 16, with higher scores indicating higher stigmatizing attitudes toward mental illness.

#### Depression and Anxiety Symptoms

Depression and anxiety symptoms will be assessed using the PROMIS depression and anxiety [44] short form scales. They both comprise 8 items, each assessing the frequency of symptoms of depression or anxiety, respectively, during the past 7 days. The depression scale focuses on negative mood (eg, feeling unhappy), decrease in positive affect (eg, nothing to look forward to), and negative views of the self (eg, feeling helpless and worthless). The anxiety scale focuses on fear (eg, fearfulness), anxious misery (eg, worry), and hyperarousal (eg, tension and nervousness). Items are rated on a 5-point scale ranging from 1=never to 5=always, and the item scores are summed to produce an overall score ranging from 8 to 40, with higher scores indicating higher symptom severity. Both short forms correlate highly with the original scales (*r*=0.96) and demonstrate validity, with the depression scale correlating with the Center for Epidemiological Studies-Depression Scale (*r*=0.81) [44] and the anxiety scale correlating with the Mood and Anxiety Symptom Questionnaire (*r*=0.80) [44].

#### General Psychological Distress

We will measure general psychological distress on the DQ5 [45]. The DQ5 comprises 5 items that ask the respondents to indicate the frequency with which they have experienced a range of distressing situations, thoughts, and feelings over the previous 30 days. The scale assesses these statements on a 5-point Likert scale ranging from 1=*never* to 5=*always*. Items include “My worries overwhelmed me,” “I felt hopeless,” and “I found social situations upsetting.” Scores are summed to produce a total score (range 5-25), with higher scores indicating more severe levels of psychological distress. The DQ5 has demonstrated high internal consistency (Cronbach α=0.86) and validity in previous studies [45,55].

#### Quality of Life

To measure the impact of the program on quality of life and for the economic evaluation, we will use the ReQoL-10 Questionnaire [46], which is a validated measure and highly suitable for measuring recovery and quality of life in the mental health context. The ReQoL-10 comprises 11 items, including 10 focused on mental health and 1 focused on physical health. The mental health items assess the frequency experienced over the last week of the thoughts, feelings, or activities mentioned, such as “I could do the things I wanted to do” and “I felt lonely,” assessed on a scale from 0=none of the time to 4=most or all of the time. Negatively worded items are reverse scored. The 11th item assesses the severity of physical health problems, including “problems with pain, mobility, difficulties caring for yourself or feeling physically unwell” experienced on a 5-point scale from 4=no problems to 0=very severe problems. The scale has been validated, with Cronbach α ranging from 0.87 to 0.92 [46,56], and has been used in economic evaluations of mental health interventions [57]. A UK-derived value set will be used to calculate utility values at each time point. The area under the curve method will be used to calculate QALYs from utility values.

#### Work and Activity Functioning

The WPAI questionnaire will be used to measure the effect of health problems on the ability of the participants to work and perform daily activities, and it will also be used for economic evaluation purposes. We altered the original WPAI questionnaire to assess days (as opposed to hours) during the past month (30 days). The original WPAI questionnaire comprises 6 questions; we will omit the first question which asks if the respondent is employed given our target group, retaining the 5 questions targeting (Question 1, Q1) the number of days missed due to health problems, (Q2) the number of days missed for other reasons, (Q3) the number of days worked, and (Q4) degree health problems affected productivity while working on 0 to 10 Visual Analogue Scale (VAS); (Q5) degree that health problems affected productivity in regular unpaid activities on 0 to 10 VAS [47]. We will derive percentage scores for absenteeism, presenteeism, work productivity loss, and activity impairment based on the predefined scoring methods [58]. Previous studies have provided support for the validity of the original scale [59].

#### Implementation Measures: Acceptability, Appropriateness, and Feasibility

To provide information regarding implementation outcomes, we will use the 3 measures designed by Weiner et al [60] that assess the acceptability, appropriateness, and feasibility of the intervention. Each of the 3 scales has 4 items assessing the acceptability (eg, “the intervention is appealing to me”), the appropriateness (eg, “the intervention seems suitable”), or the feasibility (eg, “the intervention seems easy to use”) of the program. The items are responded to on a scale ranging from 1 to 5, where 1=completely disagree and 5=strongly agree. Total scores on each scale can range from 4 to 20, with lower scores indicating a lack of each construct (acceptability, appropriateness, and feasibility). The scales demonstrate good validity, and the internal consistency is high for the 4-item scales, with Cronbach α ranging from 0.85 to 0.91 [60]. In addition, we will examine adherence levels (intervention completion), attrition, and factors associated with these indicators of engagement. Follow-up interviews (described in the Quality Assurance and Monitoring section) will also be used to inform the future implementation of the intervention in workplaces.

### Power and Recruitment

Our study’s power and sample size estimations were informed by the primary outcome measure: the level of intention to seek help from professional sources, as measured by the GHSQ. Using data from the MATES in Construction workplace trial [61], a conservative intraclass correlation of 0.02 was assumed to estimate the design effect. An RCT [62] exploring the potential effectiveness of a web-based program designed to increase help-seeking intentions for social anxiety disorder informed the anticipated difference between pre- and posttest GHSQ scores (standardized mean difference=0.2). Because the size of the workplace clusters could not be anticipated ahead of time, a conservative average cluster size ranging from 25 to 35 was assumed. We assumed 15% attrition in participant numbers from pre- to postintervention, a conservative assumption based on previous workplace trials [63] and accounting for the single-session nature of the intervention. To detect an effect size of Cohen *d*=0.2 between the intervention and comparison arms with 80% power, using a 2-tailed type I error level of 0.05, we need to recruit between 30 and 36 workplaces (assuming each workplace has 25 to 35 participants), resulting in a minimum of 900 employees.

### Quality Assurance and Monitoring

#### Overview

The project manager and research officer will be responsible for the day-to-day administration of the research. They will meet regularly (fortnightly to monthly, depending on the trial needs) with the primary investigator (PJB) to discuss the research progress. Other investigators will attend these meetings as needed, and full team meetings will be conducted several times per year throughout the project. Recruitment of trial sites and tracking of participant numbers will be monitored closely.

#### Follow-Up Interview

In the follow-up survey, participants in the active intervention condition will be asked to indicate their willingness to be interviewed regarding their experience using the program by a member of the research team after the 6-month trial period ends. Interviews will explore experiences using the program and ideas to facilitate implementation. We expect that up to 15 interviews will be required to obtain a breadth of different experiences from participants from different workplaces, work levels (eg, management and employees), ages, and genders on their experiences and views on the co-designed program. Interview participants will be selected based on this diversity. The interview will be recorded on Zoom (Zoom Video Communications, Inc) or audio recorded over the telephone for accuracy, with the audio recording only stored on secure Australian National University (ANU) IT servers. The recordings will be transcribed for analysis by a professional external transcription company. Any references to the participants’ name or workplace will be removed in transcription to reduce the chance of identification in any publications. Consent for participating in interviews, recording of interviews, and using interview data for publication will be obtained separately for the trial. Participants will be emailed an Aus $50 (US $33) e-gift card to remunerate for their time and effort participating in an interview. Participants will receive an e-gift card if they commence the interview, regardless of whether they finish it or not.

### Ethical Considerations

#### Overview

The ethical aspects of this study have been approved by the ANU Human Research Ethics Committee (protocol number 2023/053). Participants will be presented with comprehensive information regarding the study before providing informed consent on the web. Participants will not be provided with compensation for participating in the study.

Ethical issues in the literature relevant to this study relate predominantly to the potential for coercion; the use of internet-based programs; and workplace confidentiality, health, and safety concerns. These concerns include the web-based storage of data, managing privacy of personal information, obtaining consent to participate, providing opportunities to withdraw from the study, and ensuring any distressed participants in the study are provided with appropriate assistance. These issues are addressed in the following sections.

#### Data Availability, Storage, and Privacy

The major ethical issue relates to ensuring the privacy of web-based data collected and stored in password-protected computers and a secure web portal for web-based data collection and delivery of the web-based program. The website that houses the data will be assessed by ANU via a Privacy Impact Assessment to ensure it meets the privacy standards outlined in the Australian Privacy Act. Survey data will be initially stored on the custom-built secure website. After downloading it from this portal, any potentially identifying information (ie, email addresses) will be removed from the main data file and stored in a separate file. These will be linked by an ID code that will not contain any individually identifying information. We will explain to participants this is primarily so that if they wish to withdraw from the study, we can relink their data across the 2 files using this unique code and delete it.

Audio recordings and transcripts for the qualitative interviews will be stored on a secure server with access restricted to the researchers involved in the study and the IT support staff. Their interview and transcript data will be identified by a code we assign them, which will be unique to each participant but contain no identifying details (ie, a number). The audio recordings and transcripts will not be linked with the survey data and will be stored separately from the data we collected from the surveys.

We will protect the privacy of web-based data collected after downloading by storing it on secure university servers with access restricted to authorized personnel only (the project team and IT support staff responsible for managing the server). Any identifiable data associated with this project (eg, contact emails) will be deleted after the first publication is submitted. Following this, deidentified data will be retained and stored in web-based data repositories for data aggregation purposes (eg, meta-analyses) and data sharing.

The information collected from the RCT will be presented as group summaries (in aggregate form) for the purpose of publication in relevant academic journals and conference presentations. Findings will be presented with no reference to individuals. For the interview data, during the publication of results, we expect to refer to individual quotes from participants; these will be identified by broad demographic data (ie, age range, gender, and workplace type or industry only). Furthermore, individual workplaces will not be identified in any publications.

#### Participant Withdrawal and Distress

Participants will be informed that they can withdraw from the study at any time. Participants will only need to contact the researchers to withdraw if they have provided their email. If a participant requests to have their data removed from the study by sending an email to the researchers, the principal investigator (PJB) will action this request. Participants will be able to withdraw from the study at any time until the first publication is submitted, which is expected to be in early 2025. After this, we will be deleting emails and will be unable to identify an individual’s specific data. We will only recruit workplaces that have an EAP to ensure that employees experiencing mental health concerns can access effective and immediate care. We will encourage any participants who are experiencing discomfort to contact their EAP or another crisis service (eg, Lifeline). In addition, given that the purpose of the trial is to encourage help-seeking, the intervention itself will contain explicit encouragement to seek help throughout their participation in the project if they are experiencing any distress. As the program aims to improve help-seeking, our invitation letter to workplaces will clearly outline potential outcomes (such as increased utilization of EAP services) and benefits (such as enhanced mental health awareness among employees) that their participation in the trial may bring to their workplace.

#### Coercion and Informed Consent

Given that the trial will be conducted in workplaces, a critical issue is perceived coercion and pressure to participate in the study, whether explicit or implicit. Therefore, it is essential that it is made clear to the workplace key representatives, who will be coordinating the sending of invitation emails, and to the potential participants, who will be employees of the organizations, that their participation is entirely voluntary. Participants will be informed that those sending invitations to employees (eg, human resource staff and managers) will not have access to the participants’ data. In addition, workplaces will not be able to determine which of their employees have participated in the study, unless the employee wishes to tell them. We will emphasize the voluntary nature of the trial in the information and consent web-based forms for participants and explain this clearly in all communication with the workplace key representatives. Furthermore, we will discuss with each individual workplace their potential involvement in the trial and work collaboratively with them to assist them in meeting any workplace health and safety requirements relevant to participation both before and during the conduct of the trial. In addition, the workplace key representatives will be advised that we will inform our ethics committee (ANU Human Research Ethics Committee) of the details of each workplace recruited for the study to assist them in managing and supporting workplaces if any ethical concerns are raised.

Participants will be provided with a PDF version of the participant information sheet that they can save or print. The informed consent form will be provided through an information web page. The web-based consent process will require participants to answer a question indicating that yes, they wish to consent to participate in the study. Participants who do not consent can close the web page and will not proceed any further.

#### Work Health and Safety Regulations

We will engage in discussions with key representatives from workplaces regarding their potential participation in the trial. During these discussions, we will address any relevant Work Health and Safety regulations that may apply, which we will bring up when we approach workplaces to gauge their interest in participating in the trial. Furthermore, we will provide ongoing support to workplaces in navigating any potential Work Health and Safety regulation issues relevant to the trial while it is being conducted. Our letter to the key representatives in potential workplaces will explain that we will provide this support but that workplace’s will to seek their own advice on workplace health and safety requirements, as well as what may happen because of their workplace’s participation (eg, a potential increase in those seeking help from EAP services).

## Results

Recruitment of workplaces is planned to commence in April 2024. The primary and secondary outcomes, predictors of outcome, and economic analyses will be published separately after 6-month data collection is complete, which is anticipated to occur in 2025.

## Discussion

### Principal Findings

This protocol describes a cluster RCT of a new interactive co-designed on the web delivered program to increase help-seeking among adults in Australian workplaces. We expect the program will be beneficial primarily for those who experience or are at risk of experiencing symptoms of common mental health problems, such as depression and anxiety, by improving help-seeking to health services. However, it may also provide preventive education to those who may experience such problems in the future or to those who support staff experiencing mental distress. This study protocol outlines how we will test the effectiveness, acceptability, and feasibility of the intervention to increase help-seeking intentions. However, there are several potential limitations or risks that could impact the outcome of the trial, and consequent risk mitigation strategies are addressed in the following section.

### Limitations and Risks

A potential but omnipresent risk in trials is that of recruitment; this risk involves recruiting insufficient participants to enable statistical power to detect intervention effects. We expect this risk to be moderate given that the Helipad trial is situated within a large clinical trials network (Mental Health Australis General Clinical Trials Network [64]), which will provide recruitment support through an extensive network and by directly providing contacts in workplaces. In addition, based on previous trials we have conducted, we have found that recruitment is typically highly successful in web-based trials, where we have previously engaged comparable number of participants [34-36], including in cluster-based universal web-based trials [37]. It may be challenging to retain the participants; however, given the instantaneous pre- and posttest design, attrition between these 2 time points may be less likely than in previous trials. Furthermore, as outlined in our data analysis plan, this risk is somewhat mitigated by conducting statistical analyses that account for all available data (ie, mixed effects models).

Moreover, there is an ethical risk that the intervention may provide information to participants that leads them to think they may be experiencing symptoms of common mental health problems (ie, psychological distress, anxiety, or depression). We intend to measure adverse events in our postintervention and 6-month follow-up surveys, which may capture these data. However, this forms a key part of the intervention, in that it is expected to prompt those who may not know they are experiencing symptoms of a common mental disorder to seek help. There is potential that such an experience may cause distress to a small proportion of people. We expect this risk to be low, as approximately 20% (180/900) of the participants are expected to have elevated symptoms, and this is unlikely to be surprising for many of these people. Given that the intervention is specifically designed to support this group, we will be providing specific resources, including how and where to seek treatment, to equip participants to access appropriate sources of help if this occurs.

We created the Helipad web-based program with a small group of co-designers. Because the intervention was modeled around the specific views of this group, it is possible that this intervention may not be suited to all workplaces or be effective for improving help-seeking intentions in employees. The program was deliberately designed to be brief to increase engagement and uptake; however, there may be other key effective tools that could have been included but were not due to the required brevity of the program. The program as developed by the co-design group inadvertently included many aspects of other evidence-based programs for reducing stigma and increasing help-seeking [18,19,24], potentially increasing the probability the program will be found to be effective.

Co-design is a methodology that aims to actively involve people with lived experience to better align health care programs with the needs of service users [65], and funders increasingly expect that co-design principles are incorporated into research funding applications [66]. Incorporating lived experience and other stakeholder perspectives into the development of interventions and services may increase engagement and improve outcomes [67]. However, co-design is not the only solution to creating and implementing more effective interventions, and no study has compared co-designed to non–co-designed interventions head-to-head [67]. This study will test a co-designed intervention and will provide new data on the use of co-design, although the intervention contains other features that may differentiate it from the control condition, such as greater interactivity.

### Conclusions

Workplaces are ideal settings to deliver web-based mental health promotion programs to adults; however, no evidence-based programs are currently implemented widely. This study provides a significant opportunity to fill this gap by providing a rigorous test of the effectiveness of a new co-designed program in real-world settings, identifying potential barriers to and enablers of implementation, and exploring the potential economic impact of the program. If the Helipad program is found to be effective, given the web-based format and existing platform, it can be rapidly upscaled for dissemination across Australia. Furthermore, this program also provides significant potential to work with employers, insurers, and health providers to ensure all Australian workers are better equipped to seek help across the spectrum of mental health services.

## Abbreviations

A-Lit: Anxiety Literacy Questionnaire
ANU: Australian National University
D-Lit: Depression Literacy Questionnaire
DQ5: Distress Questionnaire-5
EAP: Employee Assistance Program
GHSQ: General Help-Seeking Questionnaire
GP: general practitioner
ICER: incremental cost-effectiveness ratio
PROMIS: Patient-Reported Outcomes Measurement Information System
QALY: quality-adjusted life year
RCT: randomized controlled trial
ReQoL-10: Recovering Quality of Life
SPIRIT: Standard Protocol Items: Recommendations for Interventional Trials
WPAI: Work Productivity and Activity Impairment

## Data Availability

Deidentified data will be retained for data aggregation purposes (eg, meta-analyses) and data sharing. To advance science, medicine, and public health, we may share deidentified data with any current and future funders, research projects, medical journals, or data research repositories. Some of these organizations may be located overseas. Any deidentified data that we send overseas is not protected by the Australian laws and regulations. Data will be archived for use in any related or unrelated future research with permission from the principal investigator (PJB) or his nominated delegate. Data will be shared upon reasonable request to the principal investigator (PJB) or his nominated delegate. The deidentified data will be made available for research purposes through data repositories including Health Studies Australian National Data Asset and Australian National University Data Commons [68].
